# The Antimicrobial Efficacy of *Elaeis guineensis*: Characterization, *in Vitro* and *in Vivo* Studies

**DOI:** 10.3390/molecules17054860

**Published:** 2012-04-26

**Authors:** Soundararajan Vijayarathna, Zuraini Zakaria, Yeng Chen, Lachimanan Yoga Latha, Jagat R. Kanwar, Sreenivasan Sasidharan

**Affiliations:** 1 Institute for Research in Molecular Medicine (INFORMM), Universiti Sains Malaysia, 11800 Minden, Penang, Malaysia; Email: vijaya_r_1984@yahoo.com (S.V.); latha_usm@yahoo.com (L.Y.L.); 2 Biology Program, School of Distance Education, Universiti Sains Malaysia, 11800 Minden, Penang, Malaysia; Email: zuraini@usm.my; 3 Dental Research & Training Unit, and Oral Cancer Research and Coordinating Centre (OCRCC), Faculty of Dentistry, University of Malaya, 50603 Kuala Lumpur, Malaysia; Email: chenyeng@um.edu.my; 4 Nanomedicine-Laboratory of Immunology and Molecular Biomedical Research (LIMBR), Centre for Biotechnology and Interdisciplinary Biosciences (BioDeakin), Institute for Frontier Materials (IFM), Deakin University, Waurn Ponds, Victoria 3217, Australia; Email: jagat.kanwar@deakin.edu.au

**Keywords:** antimicrobial activity, *Candida albicans*, *Elaeis guineensis*, scanning electron microscopy, transmission electron microscopy

## Abstract

The urgent need to treat multi-drug resistant pathogenic microorganisms in chronically infected patients has given rise to the development of new antimicrobials from natural resources. We have tested *Elaeis guineensis *Jacq (Arecaceae) methanol extract against a variety of bacterial, fungal and yeast strains associated with infections. Our studies have demonstrated that *E. guineensis* exhibits excellent antimicrobial activity *in vitro* and *in vivo* against the bacterial and fungal strains tested. A marked inhibitory effect of the *E. guineensis* extracts was observed against *C. albicans* whereby *E. guineensis* extract at ½, 1, or 2 times the MIC significantly inhibited *C. albicans* growth with a noticeable drop in optical density (OD) of the bacterial culture. This finding confirmed the anticandidal activity of the extract on *C. albicans*. Imaging using scanning (SEM) and transmission (TEM) electron microscopy was done to determine the major alterations in the microstructure of the extract-treated *C. albicans*. The main abnormalities noted via SEM and TEM studies were the alteration in morphology of the yeast cells. *In vivo* antimicrobial activity was studied in mice that had been inoculated with *C. albicans* and exhibited good anticandidal activity. The authors conclude that the extract may be used as a candidate for the development of anticandidal agent.

## 1. Introduction

Antimicrobial agents, particularly antibiotics, have been the standard therapy for managing microbial infections, but in recent years, genetic variation has given to pathogenic microbes a great advantage by creating antibiotic resistance so the search for new antimicrobial substances or drugs continues to be necessary. Major clinical issues arise when pathogenic microbes develop multi-drug resistance intertwined with other problems such as level of toxicity of antimicrobial drugs on host tissues. Further, reports from the scientific community have raised concerns that antibacterial drug development will not be adequately addressing the problems posed by antibiotic resistance among important bacterial pathogens [1,2,3,4]. For example, in the *First European Communicable Disease Epidemiological Report*, the European Centre for Disease Prevention and Control (ECDC) had rated antimicrobial resistance as the main factor that contributes to infectious disease in Europe due to the increase in infections owing to multidrug resistant bacteria [5]. Hospitals globally are facing the recent emergence of bacteria that are totally or almost totally resistant to currently available antibiotics is even more threatening since treatment options for infected patients are extremely limited [6,7].

The various strategies which have been identified to defeat drug resistance, the investigation of new and effective natural products exhibiting antimicrobial activity against pathogenic microorganisms is likely to play a significant role to overcome drug resistance. Malaysia, being one of the 12 mega-diversity centers of the World, is rich in all three levels of biodiversity, namely species diversity, genetic diversity and habitat diversity with many plants used for medicinal and nutritional purposes [8]. One of such plant known to have healing potential with various pharmacological activities is *Elaeis guineensis *Jacq (Arecaceae). *E. guineensis* has many therapeutic uses in traditional medicine practice. Every part of the plant can be used medicinally. The leaves of *E. guineensis* are squeezed and the juice that is obtained is placed on wounds to enhance healing [9]. The sap of this plant is also used as a laxative and the partially fermented palm wine is administered to nursing mothers to improve lactation. Fruit-husk ash is used for the preparation of a soap used for skin infections. A root decoction is used in Nigeria for headaches. The pulverized roots are added to drinks for gonorrhea, menorrhagia and as a cure for bronchitis [9]. The fruit mesocarp oil and palm kernel oil are administered as poison antidote and used externally with several other herbs as lotions for skin diseases. Palm kernel oil is applied to convulsant children to regulate their body temperature. Folk remedies of oil palm include treatment for cancer, headache and rheumatism and as an aphrodisiac, diuretic and liniment [9]. Recent studies also reported various pharmacological activity of this plant extract namely standardization of the extract, antimicrobial activity, infected wound healing activity and antioxidant activity [10,11,12,13]. Syahmi *et al*. [14] also tested the toxicity of *E. guineensis *leaf methanol extract against brine shrimp (*A. salina*) and mice*. *The results of both tests confirmed that *E. guineensis *is nontoxic and they recommended as safe natural product for commercial utilization. Hence, this work was attempted to study the potential *in vitro* and *in vivo* antimicrobial activity of *E. guineensis*.

## 2. Results

### 2.1． Antimicrobial Activity

Antimicrobial activity of leaf extract of *E. guineensis* expressed as zone of inhibition (mm) is shown in Table 1. 

**Table 1:** Antimicrobial activity of *E. guineensis*.

Microorganisms	Zone of Inhibition (mm) ^a^			Minimum Inhibitory Concentration (mg/mL)
	Leaf extract	C	M	
**BACTERIA**				
**Gram-positive**				
*Staphylococcus aureus*	**14**	**21**	**ND**	**12.5**
*Bacillus subtilis*	**12**	**22**	**ND**	**6.25**
**Gram-** **negative**				
*Escherichia coli*	**13**	**21**	**ND**	**12.5**
*Klebsiella pneumoniae*	**11**	**24**	**ND**	**>50.0**
*Pseudomonas aeruginosa*	**14**	**24**	**ND**	**12.5**
*Salmonella typhi*	**12**	**22**	**ND**	**6.25**
*Proteus mirabilis*	**13**	**23**	**ND**	**>50.0**
**YEAST**				
*Candida albicans*	**15**	**ND**	**24**	**6.25**
*Saccharomyces cerevisiae*	**-**	**ND**	**24**	**-**
**FUNGI**				
*Fusarium sp.*	**-**	**ND**	**23**	**-**
*Fusarium oxysporium*	**-**	**ND**	**22**	**-**
*Aspergillus niger*	**13**	**ND**	**24**	**12.5**
*Aspergillus flavus*	**-**	**ND**	**23**	**-**
*Microsporum canis*	**-**	**ND**	**24**	**-**
*Mucor sp.*	**-**	**ND**	**22**	**-**
*Penicillium sp.*	**-**	**ND**	**23**	**-**
*Rhizophus sp.*	**-**	**ND**	**22**	**-**
*Trichoderma viride*	**-**	**ND**	**24**	**-**
*Trichophyton mentagrophytes*	**-**	**ND**	**24**	**-**

^a^ The values ( average of triplicate) are diameter of zone of inhibition at 100 mg/mL crude extract, 30 µg/mL chloramphenicol and 30 µg/mL of miconazole nitrate. (ND: Not determined; C: chloramphenicol; M: miconazole nitrate).

The extract had great *in vitro *potential of antimicrobial activities against tested Gram-positive and Gram-negative and fungi with inhibition zone diameters ranging from 11 to 15 mm. Maximum activity was observed against *C. albicans *(15 mm) while *Klebsiella pneumoniae* (11 mm) exhibited a weak inhibition zone. The antimicrobial activity of leaf extract was also observed on the growth of filamentous fungi *A. niger *(13 mm). In contrast, the inhibition zone of solvent control methanol (negative control) was zero so that it was not active against any of the tested microorganisms. However, the two antibiotics (positive control), 30 µg/mL of chloramphenicols for bacteria and miconazole nitrate for fungi were found more effective than the leaf extract of *E. guineensis* with the inhibition zone diameters ranging between 21 and 24 mm. Based on the initial antimicrobial screening assay the strains which showed positive results against leaf extract of *E. guineensis* were selected for further studies to determine the MIC values as shown in Table 1. The MIC values against all the tested Gram-positive and Gram-negative bacteria and fungi ranged from 6.25 to 50.00 mg/mL. The MIC values indicated that the seed extract was more effective against Gram-positive bacteria at lower concentration than Gram-negative bacteria (Table 1). The lowest concentration was recorded as 6.25 mg/mL for *Bacillus subtilis, Salmonella typhi* and particularly *Candida albicans*.

### 2.2. Time Kill Study

**Figure 1:**
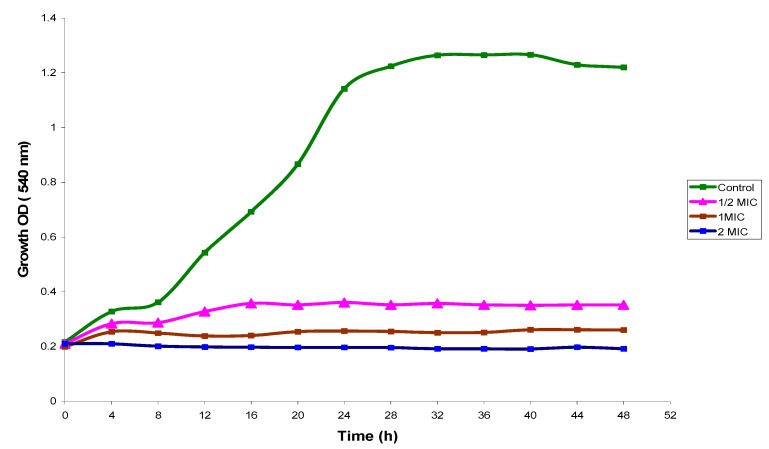
Growth profile for *Candida albicans *in Mueller-Hinton broth with 0 (Control) ½, 1 and 2 times MIC of *Elaies guineensis *leaf extract.

The growth profile curves for *C. albicans* in Mueller-Hinton Broth which being exposed to 1, ½ and 2 MIC of *E. guineensis* leaf extract over a period of 48 h are shown in Figure 1. The various MICs of seed extract shifted the normal growth profile for *C. albicans*. The control curve of the *C. albicans* demonstrated a typical microbial population growth cycle. The growth profile could be divided into three phases, from 0 to 8 h the control curve exhibited the “lag phase”, from 8 to 20 h the “exponential phase” and finally 20 to 48 h is the “stationary phase”. At ½ MIC value (3.125 mg/mL), the *E. guineensis* leaf extract demonstrated the microbial growth increases until maximum value of 0.361 OD at 16 h. At the MIC value (6.25 mg/mL), *E. guineensis* leaf extract caused a large drop in OD after 8 h, then the biomass decreased gradually and finally lead to a stationary phase where there were no visible growth changes. At 2 times the MIC value (12.5 mg/mL), *E. guineensis* leaf extract exhibited complete eradication within 2 h with a reading of 0.2 OD. 

### 2.3. *In Vivo* Antifungal Activity

**Figure 2:**
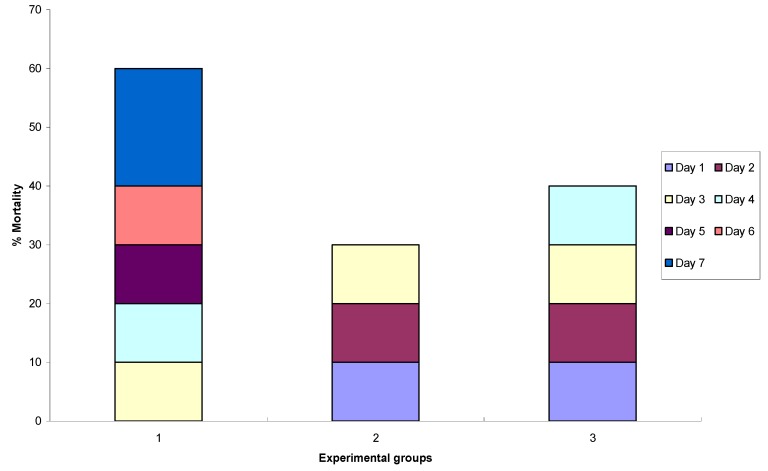
Mortality rate of mice in different group of treatment for seven days. Group 1 (control); Group 2 (treatment with ketoconazole); Group 3 (treatment with *E. guineensis *extract). *p* < 0.05.

The antifungal study was carried out with using infected mice to contemplate the effectiveness of the extract in combating infection *in vivo *(Figure 2).

Sixty percent of Group 1 animals died within 7 days of receiving phosphate buffer solution (PBS). The survival of mice was higher after ketoconazole treatment compared to other experimental groups with 70% of animals surviving to 7 days. In contrast, survival of mice with *E. guineensis *leaf extract was relatively high, with a survival rate of 60%. However, 70% of Group 2 mice receiving ketoconazole survived up to 7th day. 

**Figure 3:**
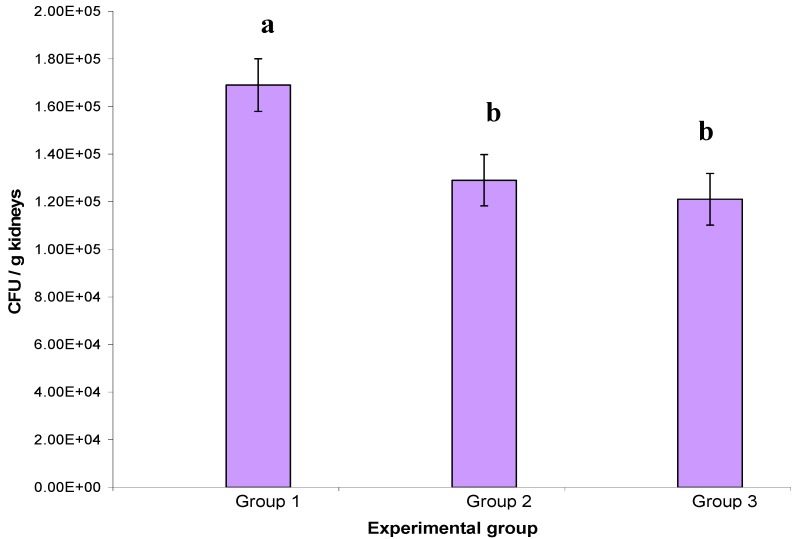
Effect of methanol extract of *E.guineensis* on *Candida albicans* recovered from kidney of mice. Group 1 (control); Group 2 (treatment with ketoconazole); Group 3 (treatment with *E. guineensis *extract). *p* < 0.05.

The extract had a good effect on the reduction of the mortality when the treatment was given after infection by the *C. albicans*, decreasing the mortality from 50% (Group 1) to 20 % (Groups 2 and 3). In addition, the effect of the extract was comparable to the commercial antibiotic ketoconazole. Figure 3 shows the mean of CFU/g of the kidney from the three experimental groups. Group 3 mice received a 2.5 g/kg body weight dose of the plant extract followed by inoculation of *C. albicans*, a significant reduction (*p* < 0.05) in CFU was observed in the studied group compared with control group.

### 2.4. SEM Observation

Scanning electron microscopy study was used to view any surface alterations and or general morphological changes of *C. albicans *cells after exposure to *E. guineensis *leaves extract. Comparisons were made between the control *C. albicans *cells and the treated *C. albicans *cells. 

**Figure 4:**
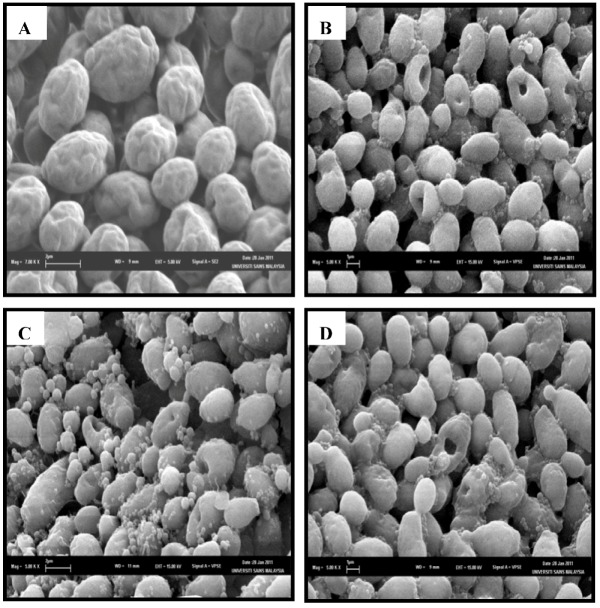
SEM micrographs of the untreated (**A**), 12 h (**B**), 24 h (**C**) and 36 h (**D**) extract treated cells of *Candida albicans*.

All the control *C. albicans* cells were generally smooth-walled bodies, spherical in shape and were mostly present in the yeast form. All the cells were lying apart showing normal budding stage (Figure 4A). After 12 h of exposure to the leaf extract of *E. guineensis*, several small invaginations and convolutions appeared on the *C. albicans *cell surfaces (Figure 4B). Other remaining cells showed a smooth surface as observed in control cells. More invaginations and convolutions appeared in the 24 h treated cells (Figure 4C). Cracks in the cell wall were detected in the last group, which was treated with the leaf extract for the duration of 36 h (Figure 4D). Thus, it was assumed at this stage that the cells had completely lost their metabolic functions.

### 2.5. TEM Observation

**Figure 5:**
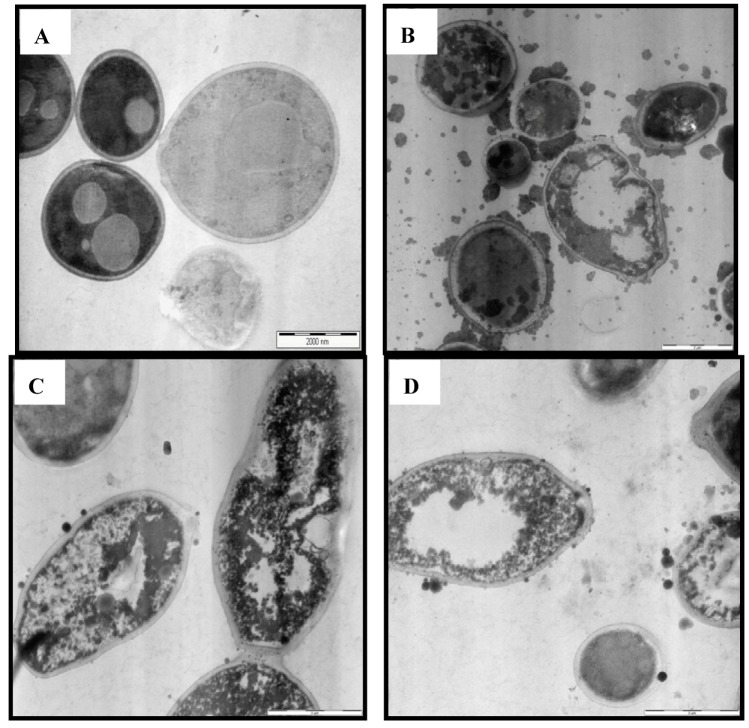
TEM micrographs of the untreated (**A**), 12 h (**B**), 24 h (**C**) and 36 h (**D**) extract treated cells of *Candida albicans*.

The scanning electron microscopy studies results were compared with those seen on the yeast cells by means of transmission electron microscope. It was found that the untreated (control) *C. albicans *cells as examined by TEM showed typical *C. albicans *morphology with a uniform central density (Figure 5A). Control cells typically had a structured nucleus and a cytoplasm with several elements of endomembrane system enveloped by a regular, intact cell wall plasma membrane lying closely to the cell wall. After being exposed to *E. guineensis *extract for 12 h (Figure 5B), the *C. albicans *cells appeared to possess large vesicles while membranous bodies were found disposed within the cell. On the other hand, the cell walls of the 24 h treated cells (Figure 5C) appeared vertically more oblong accompanied by the shrinkage of protoplast and disruption of cytoplasmatic membrane. In addition, the cytoplasmic volume is contemplated decreasing following the cell membrane invagination causing notable structural disorganization within the cell cytoplasm. It can be deduce that the leaf extract had triggered cell membrane to become dysfunctional. The significant effect of the extract can be viewed after 36 h of treatment (Figure 5D). All the inner organelles are completely discomposed while cell membranes appear undulant. Yeast cells were found collapsed.

### 2.6. Identification of Antifungal Bioactive Compound (s)

#### 2.6.1. FTIR Analysis

**Figure 6:**
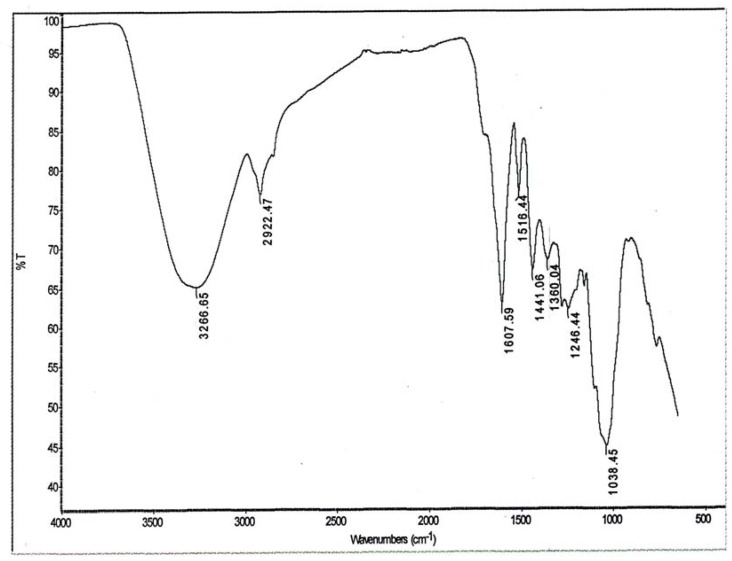
Fourier transform infrared (FTIR) spectroscopy analysis obtained for the leaf extract of *Elaeis guineensis*.

FTIR spectroscopic studies were carried out to investigate the possible antimicrobial and antifungal agents that might present in the extract of *E. guinensis* (Figure 6). The spectra of extracts were recorded in the form of an interferrogram to which results of various functional groups were exhibited between the wavelength of 100–1,700 cm^−1^ and 2,900–3,500 cm^−1^. Eight functional groups were found. The spectrum denoted a broad at 3,406.05–3,436.91 cm^−1^ which is assigned to (OH) stretching vibrations from phenols present in the extract. Strong to medium intensities broad bands are observed at 2,983.67–2,870.84 cm^−1^ which suggest energetically favored carboxylic acid groups. Other bands are observed to be appearing at 1,514.00–1,450.00 cm^−1^ and 1,038.45 cm^−1^ are attributed to the existence of aromatic, alkene C=C, primary alcohol and phenol groups, respectively.

#### 2.6.2. Gas Chromatography-Mass Spectrometry (GC-MS) Analysis

**Figure 7:**
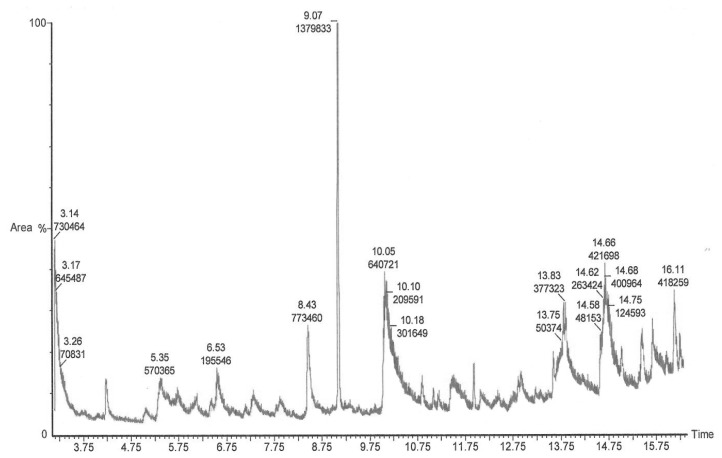
Gas chromatogram of *E. guineensis *methanolic extract.

GC-MS analysis was done to identify the compounds responsible for observed antimicrobial activity. The gas chromatogram of the methanolic leaf extract of *E. guineensis *showed eleven compounds (Figure 7). The retention times (Rt) were reported in minutes. The eleven major compounds identified in the methanolic extract were dimethyl sulfoxide, which was eluted twice with different retention times (Rt 3.14; 3.17); tetrahydro-*trans*-3,4-Furandiol (Rt 5.35); 1-Amino-2,6-dimethylpiperidine (Rt 6.53); 2,3-dihydro-3,5-dihydroxy-6-methyl-4*H*-Pyran-4-one (Rt 8.43); 4-methyl-1-(1-methylethyl)-3-Cyclohexen-1-ol (Rt 9.07); 5-(hydroxymethyl)-2-Furancarboxaldehyde (Rt 10.05); D-mannose (Rt 13.83); 1,6-anhydro-α-D-glucopyranose (levoglucosan) (Rt 14.66); 3-*tert*-butyl-4-hydroxyanisole (Rt 15.42); 3,4-dihydro-2(1*H*)-isoquinolinecarboximidamide (Rt 15.65); and 5-isopropenyl-2-methylcyclopent-1-ene-carboxaldehyde (Rt 16.11).

## 3. Discussion

In this study, we investigated the *in vitro* and *in vivo* activity of leaf extract of *E. guineensis*. The *in vitro* activity was tested against a wide panel of clinical isolates including bacterial strains, as well as fungaland the yeast *C. albicans*. The results indicate a broad spectrum activity of the leaf extract of *E. guineensis*, which was more effective against Gram-positive bacteria than Gram-negative bacteria (Table 1). This occurrence could be explained by that the structures of cell envelope differ significantly between Gram-positive and Gram-negative bacteria. Gram-negative bacteria possess an outer membrane surrounding the cell wall, which restricts diffusion of hydrophobic compounds through its lipopolysaccharide covering. The cell wall of Gram-positive bacteria without this outer membrane can be permeated more easily and the constituents of *E. guineensis* extract can disturb the various molecular targets including the cytoplasmic membrane, proton motive force (PMF), electron flow, active transport and coagulation of cell contents [15]. This finding was further verified by our SEM and TEM study which indicated the outer physical changes that took place on the fungal cells structures while the TEM describes the inner cell morphology and cytology changes that further substantiate the aptitude of *E. guineensis* as a potential anticandidal compound (Figure 4 and Figure 5).

Time killing profile was applied to further corroborate the *in vitro* antimicrobial results observed. The time killing study exhibited a prolonged anti-candididal activity when *Candida albicans* cells were exposed to the methanolic extract of *E. guineensis* at ½ MIC, MIC and 2 MIC for 48 h. *In vitro* antimicrobial methods are controversial because the results do not always correlate with the *in vivo* conditions or clinical outcomes. Such a fact clearly shows that traditional MIC determinations are inadequate for determining an antimicrobial agent’s clinical effectiveness against pathogens. Time-kill curves of pathogens exposed to several extract concentrations measure antimicrobial activity over time, and may reveal differences in activity between agents at different MIC concentrations. Our results indicate that *E. guineensis* extract possesses a good anti-yeast activity; concentration dependent killing was observed against the strains tested. 

The results obtained with *in vitro* assays encouraged us to exploit the *in vivo* potential of *E. guineensis* extract against lethal candidal challenge in animals. The use of an *in vivo* model in mice was suggested by the previous results of *in vivo* oral toxicity tests that indicated a much better LD_50_ for oral administration of these *E. guineensis* extracts. The *in vivo* results using the *E. guineensis* extract is encouraging and parallel those obtained *in vitro*. *E. guineensis* extract was highly protective against *C. albicans* infection, with 80% protection at respectively 2.5 g/kg. Notably, protection is achieved at *E. guineensis* doses used in this study, suggesting a satisfactory therapeutic index for this extract. In keeping with the *in vitro* results, *E. guineensis* also defends mice from lethal i.v. dose of *C. albicans*, suggesting that *E. guineensis *extract are effective *in vitro* than *in vivo*. The *in vivo *results so far obtained indicate that, the effect of *E. guneensis *extract was significantly comparable to the commercial antibiotic ketoconazole.

Our results indicated that *E. guneensis *extract had good antimicrobial activity towards the tested pathogenic microbes. Then, the extract was subjected to FTIR and GC-MS analysis, which revealed the presence of antimicrobial compound and with various functional groups. Both the 3,406.05–3,436.91 cm^−1^ and 1,038.45 cm^−1^ absorption bands were attributed to (OH) stretching vibrations from phenols, a class of chemical compounds containing hydroxyl functional groups (–OH) attached to an aromatic hydrocarbon group. Numerous recent studies have reported that phenolic compounds from natural resources display antifungal activity. Duke reported that the common herbs tarragon and thyme both possessed a phenylpropane-derived compound (phenol) which is very active against fungi. The location site(s) and the amount of hydroxyl groups found on the phenols are related to their relative toxicity towards microorganisms with evidence that increased hydroxylation results in increased toxicity [16]. Likewise, it was also reported that more highly oxidized phenols show more inhibitory activity [17,18] which includes enzyme inhibition by the oxidized compounds, possibly through reaction with sulfhydryl groups or through more nonspecific interactions with the proteins [19]. Literature searches also reveal that phenolics also serve as an antimicrobial agents [20,21,22] with activity that includes adsorption and disruption of microbial membranes, interactions with enzymes and substrates and metal ion deprivation [23,24]. 

Strong to medium intensity bands were observed at 2,983.67–2,870.84 cm^−1^ confirming the presence of carboxylic acid functional groups. Carboxylic acids are essential groups found in other important plant secondary metabolites such as olean-27-carboxylic acid, a type of triterpene reported to shows antibacterial activity [25]. Likewise carboxylic acids were found to be linked with many antimicrobial and antifungal activities which are found to exist in various plant metabolite molecular structures such as ursolic acid which had been reported as a strong antibacterial agent [26]. In other cases, Jabeen *et al.* [27] have mentioned that the elimination of fungal pathogens by the seed extract of *Moringa oleifera* can be attributed to the presence of carboxylic acids*. *This fact is further substantiated by Shittu *et al. *[28] who had earlier reported on carboxylic acids being responsible for the antimicrobial activity in *Sesame radiatum. *Other bands being detected in the FTIR analysis appeared at 1,514.00–1,450.00 cm^−1^ and 1,038.45 cm^−1^ that correspond respectively to the presence of C=C aromatic, alkenes, primary alcohol, and again phenol groups. Plants produce vast and diverse numbers of secondary metabolites that include these active groups. Other chemical components of the extract also certainly could contribute although lack of chemical profiling has never been reported on this. 

In addition, GC-MS analysis also revealed the presence of several potentially antimicrobial components in the *E. guineensis* extract. The antimicrobial properties of *E. guineensis* extract are suspected to be associated with the dimethyl sulfoxide, 2,3-dihydro-3,5-dihydroxy-6-methyl-4*H*-pyran-4-one, 5-(hydroxymethyl)-2-furancarboxaldehyde, and D-mannose, which was detected by the GC-MS analysis in this study. All these components have been tested previously and reported to have a significant antimicrobial activity [29,30,31,32]. It is possible that these compounds are mainly responsible for the antimicrobial effects observed in this study.

## 4. Experimental

### 4.1. Plant Collection

Fresh leaves of *E. guineensis* were collected Semeling, Kedah in January 2010 and authenticated at the Herbarium of the School of Biological Sciences, Universiti Sains Malaysia, Pulau Pinang, Malaysia where a sample (voucher number 11037) has been deposited. The leaves were separated and cut into small pieces, which were first washed with tap water and then with distilled water. The leaves were then dried in an oven at 60 °C for 7 days, after which the dried leaves were ground into a fine powder using a grinder and stored in clean, labeled airtight bottles.

### 4.2. Preparation of Plant Extract

Dried sample (approximately 100 g) was added to methanol (300 mL) and soaked for 4 days at room temperature (30 ± 2 °C). The suspension was stirred from time to time to allow the leaf powder to fully dissolve in the methanol. Removal of the sample from the solvents was done by filtration through cheesecloth followed by filter paper (Whatman No. 1); the filtrate was concentrated under vacuum (vacuum pressure: 500 N/m^2^) at 40 °C [33] to one-fifth its volume using a rotary evaporator and then sterilized by filtration using a 0.22-mm membrane for antimicrobial assay. The thick paste obtained was further dried in an oven at 40 °C. The resultant extract was kept at 4 °C for further analysis.

### 4.3. Determination of the Antimicrobial Activity

#### 4.3.1. Antimicrobial Disc Diffusion Assay

Antimicrobial and antifungal activities of *E. guinensis *extract were investigated using the disc diffusion method [34,35]. The test microbes were removed aseptically with an inoculating loop and transferred to a test tube containing 5 mL of sterile distilled water. Sufficient inoculums were added until the turbidity became equaled to 0.5 McFarland (10^6^ colony-forming units (CFU)/mL of bacteria or 2 × 10^5^ (CFU)/mL fungi cells/spore) standards (bioMerieux, Marcy Petoile, France). The test tube suspension (1 mL) was added to 15 to 20 mL of Mueller-Hinton Agar and Sabouraud Dextrose Agar before setting aside the seeded agar plate (9 cm in diameter) 15 min to solidify. Three Whatman’s filter paper No.1 discs of 6 mm diameter were used to screen the antimicrobial activity. Each sterile disc was impregnated with 20 μL of the extract (corresponding to 100 mg/mL of crude extract), chloramphenicol or miconazole nitrate (30 µg/mL, as positive control for bacteria and yeast/fungus respectively) and 80% methanol (v/v) (as negative control) after the discs were placed on the surface of the seeded plates. The plates were incubated in the incubator (Memmert, Schwabach, Germany) at 37 °C and at 28 °C for yeast/fungi. The zones of inhibition around the discs were measured after 18 to 24 h of incubation for bacteria, and 48 to 96 h for fungi. Sensitivity of the microorganism species to the plant extract was determined by measuring the sizes of inhibitory zones (including the diameter of disc) on the agar surface around the discs, and values less than 8 mm were considered as not active against microorganisms [36]. All the microbial strains used in this study were local clinical isolates. All of the experiments were performed in triplicate and results were reported as the average of three experiments.

#### 4.3.2. Minimum Inhibitory Concentration (MIC) Determination

Due to the display of significant activity by the *E. guinensis *leaf extract against the tested microorganisms, further determination of the minimum inhibitory concentration was investigated. Two-fold broth dilution method was implied as described by [37] with slight modification. The plant extract (500.00 mg) was dissolved in distilled water (10 mL) to reconstitute an extract solution of 50.00 mg/mL as stock. Subsequently, a serial dilution technique was carried out with 2.5 mL of stock solution being transferred to a test tube containing 2.5 mL nutrient broth medium to give a concentration of 25.00 mg/mL. Next, 2.5 mL of solution from the first test tube was transferred into another a second test tube containing nutrient broth medium that gave rise to a concentration of 12.50 mg/mL and similarly technique was continued until a final concentration of 0.098 mg/mL was achieved. 

The test microbes were removed aseptically with an inoculating loop and transferred to a test tube containing 10 mL of sterile distilled water. Sufficient inoculums was added until the turbidity was equivalent to 0.5 McFarland (10^6^ CFU/mL) standard (bio-Meriuex, Marcy Petoile, France). An inoculum size of 0.5 mL bacteria/fungal was added to each test tube by maintaining the final concentration of the extract in each test tube. After 18 h of incubation at 37 °C, the tubes were examined for bacterial growth. Growth was observed in those tubes where the concentration of the extract was below the inhibitory level where the broth medium turned into turbid or looks cloudy. The MIC value of the extract was taken as the lowest concentration that showed no growth or non-turbid in the test tube [38].

### 4.4. Time-Kill Study

The time killing study was conducted with ½, 1 and 2 times MIC over time whereby a growth profile curve was plotted [39]. A 16 h culture was harvested by centrifugation, washed twice with phosphate saline and re-suspended in phosphate saline. The suspension was adjusted using the McFarland standard and was then further diluted in phosphate saline to achieve an approximation of 10^7^ colony forming unit (CFU/mL). Leaf extract of *E. guineensis *was added to aliquots of 25 mL Mueller-Hilton broth (MHB) in 50 mL Erlenmeyer flask and was placed in a water bath at 37 °C with amounts corresponding to the concentration of ½ ,1 and 2 times of MIC value (6.25 mg/mL) upon the addition of the inoculums. Free medium without extract was used as a control. Next, 1 mL of *C. albicans *inoculum was added to all Erlenmeyer flasks. After the addition of the inoculums 1 mL portion was removed from Erlenmeyer flask and the growth of *C. albicans *was monitored using this portion by measuring the Optical Density at 540 nm 9 UV-9100, Ruili Co., Beijing, China). The growth of *C. albicans *was measured every 4 h throughout 48 h by the above method.

### 4.5. *In Vivo* Antifungal Assay

#### 4.5.1. Laboratory Animals

Swiss albino mice (male) weighing between 25 and 35 g were used. The cages with the mice were placed in a room (temperature 26 ± 2 ^ο^C) with controlled cycles of 12 h of light and 12 h of darkness; light went on at 7 am and relative humidity was 45–55%. Water and food were provided to animals *ad libitum*. The experimental protocols were approved by the Institutional Animal Ethics Committee (IAEC) at Universiti Sains Malaysia. Experiments were conducted in accordance with the internationally accepted principles for laboratory animal use and care (EEC Directive of 1986; 86/609/EEC).

#### 4.5.2. Antifungal Assay

Standard intravenous (i.v.) inoculation of *C. albicans *was used in this study where 1 × 10^7^ viable cells/mL PBS, of which 0.1 mL was injected into the lateral tail vein of mice [40]. Animals were divided into three groups of 10 mice each and received treatment as described in Table 2. All mice were killed by cervical dislocation on day 5 after i.v. *C. albicans *inoculation. The drug and extract concentration for *in vivo* model were determined based on the body weight of the mice used in this study (drug weigh/ kg body weight of mice). The kidneys of each animal were removed aseptically, and 0.1 mL of blood was withdrawn from the renal artery and 0.1 mL of heparin (25 U/mL), as an anticoagulant was added into the blood sample. The kidneys were then, placed in sterile centrifuge tubes and homogenized in 5 mL of sterile PBS. Aliquots from each homogenate and blood samples were serially diluted, plated on Sabouraud dextrose agar plates, and incubated at 37 °C for 24 h. All cultures were done in triplicate. The colonies were then enumerated and the colony forming units (CFU) were calculated per gram of organ and per mL of blood sample, respectively. 

**Table 2:** Animal experimental groups and received treatments data summary.

Group	Treatment
Group 1 (Negative control)	i.v. *Candida* 24h gap, follow by treatment with PBS (o.a. once daily for 7 days)
Group 2 (Positive control)	i.v. *Candida* 24h gap, follow by treatment with ketoconazole, 10 mg/kg body weight (o.a. Once daily for 7 days)
Group 3 (curative)	i.v. *Candida* 24h gap, follow by treatment with *E. guineensis* extract, 2.5 g/kg body weight (o.a. once daily for 7 days)

i.v.: intravenous; o.a.: oral administration; PBS: phosphate buffer saline.

### 4.6. Scanning Electron Microscope (SEM) Observation

Scanning electron microscope (SEM) observations were carried out on *C. albicans *cells. One milliliter of the *C. albicans *cell suspension with the concentration of 1 × 10^6^ cells per mL was inoculated on four Sabouraud dextrose agar plate and then incubated at 30 °C for 12 h. Two milliliter of 6.25 mg/mL, *E. guineensis *leaf extract was dropped into three inoculated agar and further incubated for another 12, 24 and 36 h at the same incubation temperature. An 80% methanol (v/v) treated culture was used as a control. A small block of yeast containing agar was withdrawn from the inoculated plate 0, 12, 24 and 36 h and fixed for scanning electron microscope (Leo Supra 50 VP Field Emission SEM, Carl Zeiss, Oberkochen, Germany) [41]. The SEM study was done under the following analytical conditions: L = SE1, WD = 21 mm, and EHT = 10.0 kV to study the effect of extract on *C. albicans* cells. 

### 4.7. Transmission Electron Microscope (TEM) Observation

Transmission electron microscope (TEM) observations were carried out on *C. albicans *cells. The preparation of the cells on the plates was as Section 3.4.4. After incubating the 0, 12, 24 and 36 h plates, all were fixed for TEM observation [42]. TEM analyses was performed on samples fixed in Mc-Dowell-Trump fixative prepared in 0.1 M phosphate buffer, rinsed in buffer and postfixed for 2 h in 1% osmium tetroxide prepared in the phosphate buffer. The sample in agar was prepared and dehydrated in ethanol and finally embedded in resin. The resin with the embedded *C. albicans *cells were cut into ultra-thin sections in the ultramicrotomy process. Finally the ultra thin sections were stained with 2% uranyl acetate and lead citrate, and observed under TEM (Philip CM12, Eindhoven, Netherlands). 

### 4.8. Identification of Antifungal Bioactive Compound(s)

#### 4.8.1. FTIR Analysis

The methanol extracts of *E. guineensis* was mixed with potassium bromide (KBr) using a mortar and pestle, and compressed into a thin pellet. Infrared spectra were recorded as KBr pellets on a Schimadzu FTIR Spectrometer 8000 series (Columbia, WA, USA), between 4,000 and 500 cm^−1^. All determinations were performed in triplicate**.**

#### 4.8.2. Gas Chromatography-Mass Spectrometry (GC-MS) Analysis

The GC-MS analysis was done on a thermo gas chromatography mass spectrometer (model Shimadzu 2010, Tokyo, Japan) equipped with DB-5 capillary column (30 m long, 0.25 mm i.d., film thickness 0.25 µm). The column temperature program was 50 °C for 6 min, with 5 °C increases per min to 250 °C; which was maintained for 30 min. The carrier gas was helium at a flow rate of 1 mL/min. The detector and injector temperatures were both maintained at 250 °C. The quadrupole mass spectrometer scanned over the range 28–400 amu at 1 scan/ sec, with an ionizing voltage of 70 eV, an ionization current of 150 Ma and an ion source temperature of 200 °C. To determine the Kovats index of the components, a mixture of alkenes (C9-C24) was added to the extract before injecting in the GC-MS equipment and analyzed under the same conditions as above. The compounds were identified by computer searched in the commercial libraries on NIST (National Institute of Standard and Technology) and by their Kovats retention indexes [43]. 

### 4.9. Statistical Analysis

One*-*Way ANOVA was used to compare the means of three experimental groups with Tukey’s post*-*hoc test to calculate least significant differences. The difference between means was considered significant when *p* was <0.05.

## 5. Conclusions

In conclusion, this study provides new scientific information about *E. guineensis*, based on its antimicrobial potential and chemical profiling that has never been reported. The anticandidal activity of *E. guineensis* may be attributed to the various phytochemical constituents present in the extract. The purified components may have even more potency with respect inhibition of microbes. Further work on the types of phytoconstituents and purification of individual groups of bioactive components could reveal the full potential of the *E. guineensis *extract to inhibit several pathogenic microbes and encourage in the developing a novel broad spectrum antimicrobial herbal formulation in the future, especially against *C. albicans*. 
